# Development of small blood volume assays for the measurement of oxidative stress markers in mammals

**DOI:** 10.1371/journal.pone.0209802

**Published:** 2018-12-27

**Authors:** Evan Langille, Vincent Lemieux, Dany Garant, Patrick Bergeron

**Affiliations:** 1 Bishop’s University, Sherbrooke, Québec, Canada; 2 Chemistry Department, Memorial University of Newfoundland, St. John’s, NL, Canada; 3 Département de biologie, Université de Sherbrooke, Sherbrooke, Québec, Canada; Tufts University, UNITED STATES

## Abstract

Measuring oxidative stress has become increasingly valuable in ecological studies, especially when different markers are measured on the same individual. However, many of the current methods lack sensitivity for analysis of low blood volume samples, which represent a challenge for longitudinal field studies of small organisms. Small blood volumes can usually only be analysed by using a single assay, therefore providing limited information on individual’s oxidative profile. In this study, we used blood collected from a population of wild eastern chipmunks (*Tamias striatus*) and modified methods presented in the literature to improve analytical selectivity and sensitivity required for small blood volumes. Specifically, we proposed a modified malondialdehyde (MDA) analysis protocol by HPLC and also optimized both the uric acid independent ferric reducing antioxidant power (FRAP) and hypochlorous acid shock capacity (HASC) assays. Development of the three modified methods was achieved with a sensitivity and repeatability that meets standards of field ecology while allowing measurement of all three assays in duplicate using less than 60 μL of plasma. Availability of these tests using small blood volumes will provide ecologists with a more comprehensive portrait of an individual’s oxidative profile and a better understanding of its determinants and interactions with the environment.

## Introduction

Measuring markers of oxidative stress has become increasingly popular and useful for the evaluation of individual’s physiological states. Previous work on humans reported that abnormally high oxidative stress can be associated with a number of diseases [1–3] and increased aging at cellular and organismal levels [4]. In ecological studies, oxidative stress is increasingly used to identify the factors that may cause physiological stress and study their potential involvement in life-history trade-offs [5]. Measuring several markers of oxidative stress has now become the norm to better understand an individual’s oxidative profile because of the complexity of its regulation mechanisms [6–8]. However, using multiple oxidative stress markers and estimate methodological precision with replicate measurements usually requires large blood sample volumes, making it difficult for researchers to perform comprehensive analyses of oxidative stress profile of small wild animals without being invasive.

Our goal in this study was to adapt published methodologies of three commonly reported markers of oxidative profile in order to decrease the blood volume required to quantify: i) malondialdehyde (MDA) concentration, ii) the ferric reducing/antioxidant power (FRAP) assay, and iii) the hypochlorous acid shock capacity (HASC) assay [9–12]. We first introduce these three markers and some of their current main limitations. Then, we present in-house assays optimized to yield the most selective analysis for each marker using the smallest blood volumes possible.

### Oxidative damage

#### Malondialdehyde (MDA)

Quantifying levels of malondialdehyde (MDA) is performed to assess lipid peroxidation in a wide range of disciplines from food preservation [13] to medicine [14]. In ecological studies, plasma MDA levels are commonly used to estimate the overall oxidative damages experienced by an individual [5,7,10,15]. Traditionally, MDA levels were quantified using the thiobarbituric acid reactive substances (TBARS; [16]), an assay that is still commonly used in animal physiology [17–19]. Thiobarbituric acid reacts with tissues and create a colored adduct that can be quantified by spectrophotometry.

Although being a relatively simple assay, TBARS has been readily criticized for its lack of specificity to MDA. Thiobarbituric acid can react with a wide range of components present in the plasma, such as aldehydes, sugars and urea [20–22]. Thus, external factors like diet or nitrogen elimination can confound assay values, leading to an overestimation of MDA levels up to 3 folds [23,24]. A greater specificity to MDA can be achieved with the TBARS assay by injecting the reacted sample into a high-performance liquid chromatograph (HPLC) [15,23,25,26]. However, HPLC methods for plasma MDA detection are mainly developed in biomedical studies where blood sampling volume is not a limiting factor, requiring plasma volumes ranging from 50 μL to 100 μL [23,27,28]. Therefore, approximately 200–400 μL of whole blood would have to be collected from an individual to enable replicated measurements of MDA levels. This amount of blood may require sampling procedures that are too invasive for conservation studies or for longitudinal studies of smaller species, preventing the measurement of multiples assays on a single sample [5].

Also, the MDA generated in the cell quickly binds to amino acids and proteins due to its high reactivity, making it present in both free and bound states in tissues. Free MDA readily reacts with TBA while bound MDA must return to its free form before reacting. Although most bound MDA becomes free during the derivatization process [27], it appears crucial to thoroughly liberate all the bound MDA since it may represents about 90% of all the MDA present in a sample [26]. Adding an additional alkaline hydrolysis step allows a more complete and uniform release of bound MDA, thus yielding better measurements [26,29].

Moreover, high-throughput methods can be necessary for larger-scale studies, since MDA levels in stored plasma samples [25] or processed samples [27,30] can degrade over time. Therefore, optimizing the retention of the compound while using the shortest possible runtime could allow to process hundreds of injections per day on a single HPLC system.

### Antioxidant protection

#### Ferric reducing/antioxidant power (FRAP)

The ferric reducing/antioxidant power (FRAP) assay has been used extensively in various disciplines ranging from agronomy and nutrition [31,32] to ecological studies [8,9,17]. Antioxidants in the samples reduce ferric-tripyridyltriazine (Fe^3+^-TPTZ) complex to ferrous tripyridyltriazine (Fe^2+^-TPTZ), producing a blue colour. The coloration intensity is proportional to the reducing capacity of the mainly non-enzymatic antioxidants in the plasma, hence providing the overall antioxidant capacity. The FRAP assay is a simple, quick and inexpensive test which already requires fairly small blood volumes [17,19,33,34].

As for other antioxidant capacity test like the TAS/TEAC assay [35], FRAP values are strongly correlated with uric acid levels, which could bias the antioxidant power estimate [33]. It may be difficult to decipher whether fluctuations in uric acid levels are due to other incidental metabolic processes, like an increase of amino acid catabolism, or a change in antioxidant regulation [35,36]. Furthermore, differential selective pressure among species may have operated to induce variation in concentration and dynamics of uric acid [36]. In recent studies, its level is often assayed in parallel with antioxidant assays and statistically accounted for [37,38]. However, this approach requires the use of an extra blood assay kit which may add measurement errors to the data and reduce the amount of blood available to other assays.

An approach to remove uric acid from a plasma sample has been previously proposed by pre-treating the plasma with great excess of uricase to specifically eliminate uric acid [39]. This approach spares the need of using an extra blood assay kit but high plasma volumes are usually required (e.g. 200 μL; [40]. Here we optimized the amount of uricase added to fully remove all uric acid from the samples and we modified the incubation and assay protocol for smaller blood volumes.

#### Hypochlorous acid shock capacity assay (HASC)

In biological studies, the HASC assay is commonly referred to by its kit name OXY-adsorbent test^TM^ from Diacron International (Grosseto, Italy). This total antioxidant test is popular in animal ecological studies [5,41] and it is also used in agronomy [42,43] and neurophysiology [44]. The test works by incubating a plasma sample with an excess of hypochlorous acid, a strong oxidant. The hypochlorous acid oxidizes any reduced components in the plasma, then a chromogenic dye is added to quantify the remaining hypochlorous acid. The original concentration of hypochlorous acid minus the remaining quantity provides the total antioxidant capacity of the sample. The HASC assay is not correlated with uric acid levels [11]. Because of its general mechanism and the non-specificity of the pro-oxidant reagent, this assay allows most of the antioxidant mechanism to mitigate plasma oxidation. However, hypochlorous acid is not the substrate of the antioxidant enzymes, which are not contributing to its neutralisation. Therefore, the HASC assay measures total non-enzymatic antioxidant capacity. Comparative studies between HASC and FRAP have found no correlation [11] or weak positive correlation [45], suggesting that the two assays are complementary and may generate a more complete picture of the total antioxidant capacity when used together [11,45].

OXY-adsorbent test^TM^ requires small plasma/serum volumes (10 μL) which allows to simultaneously measure other oxidative stress or physiological components. However, with large sample sizes and/or longitudinal studies, these kits can prove to be expensive. Because of the simplicity of the assay, we propose a low cost in-house version of this assay that is specifically adapted for small plasma volumes.

## Materials and methods

### Study population and sample collection

In order to develop methods using low blood volume often available in ecological studies, we used a population of wild eastern chipmunks (*Tamias striatus*) already followed as part of a longitudinal study in southern Québec, Canada (45°05’ N, 72°26’ W) and on which only small amount of blood can be collected (e.g. less than 200 μL of whole blood,[10]. Since 2012, individually marked chipmunks from three sites (6.8 ha, 6.8 ha and 3.2 ha) are systematically trapped using Longworth traps from May to September and monitored for phenotypic traits such as behavior, body mass, sex, reproductive status and parasite load (see [46] for details). We assess the methods’ ability to provide reliable and repeatable results for individuals from this population to ensure that the methods are accurate enough to provide a high level of measurable variability among individuals.

The current study took place between June 21 and June 29 in 2017 when chipmunk activity was high. Upon capture, chipmunks were gently constrained in a handling bag and blood was collected in 70 μL heparinized glass capillary tubes (Fisher Scientific, ON, Canada) after clipping the tip of a toenail following [10]. Capillaries were immediately stored at 4 ^o^C upon collection and were processed within 5.25 hours by centrifugation of the tubes at 12 000 RPM and 4 ^o^C for 6 min in a microhematocrit centrifuge (M24, LW Scientific, Lawrenceville, GA). A metal file was used to cut between the buffy coat layer and the plasma from the capillary, and positive pressure of a syringe was used to push the sample into 0.5 mL microcentrifuge tubes. Blood fractions from each capillary were kept in separate microcentrifuge tubes. The tubes were stored at -15 ^o^C for up to 5 days, before being transported to -80 ^o^C for storage for up to 7 months.

We collected blood on 69 different individuals. However, because there was variation in the amount of blood available per chipmunk, 18 individuals were measured on one marker, 34 on 2 markers and 17 of all three markers. Ethics approval was obtained from both The Canadian Council on Animal Care (#A2016-01—Bishop’s University) and the Ministère des Ressources naturelles et de la Faune du Québec (#2017-05- 01-102-05-S-F).

### MDA analysis by HPLC

#### MDA reagents and system settings

HPLC grade acetonitrile was purchased from VWR Int. (Mississauga, ON, Canada). A license to procure and possess 2-thiobarbituric acid was obtained from Health Canada (authorization #43305.06.17). The 2-thiobarbituric acid was purchased from EMD Millipore (Billerica, MA, USA). Malondialdehyde bis (diethylacetal), butylated hydroxytoluene, trichloroacetic acid and trifluoroacetic acid were purchased from Sigma-Aldrich (Oakville, ON, Canada). All other reagents used were of analytical grade unless otherwise noted. Ultrapure water was made in-house using a Barnstead NANOpure water system (Resistivity ≥ 18.2 MΩ • cm^-1^).

MDA was determined using an Agilent Technologies 1100 HPLC system consisting of an autosampler (G1313A), a binary pump (G1312A), a thermostatted column compartment (G1316A) and a variable wavelength detector (G1315A) (Santa Clara, CA, USA). The “OpenLab Chromatography Data System” software (Agilent Technologies) was used to control the instrument and collect the data. Analytical separations were performed using a Varian ODS2 250x4.6 mm column at 30.0 ^o^C. The mobile phase was an isocratic mixture of 0.1% TFA in water: acetonitrile (70:30 V/V) at a flow rate of 1.00 mL min^-1^. The backpressure of the system was 165±5 BAR. The injection volume was 20 μL. Detection was at a wavelength of 532 nm. The runtime of the method was 4 min and the retention time of the TBA_2_-MDA adduct was 3.00±0.05 min.

#### MDA analysis for 20 μL of plasma

The MDA quantification protocol was adapted from [23]. We added 2 μL of butylated hydroxytoluene (0.1M) in anhydrous ethanol and 5 μL of NaOH (2.0 M) to a 20 μL aliquot of plasma or standards (see below), before being placed in a shaking dry block incubator set at 60 ^o^C for 30 min and 400 RPM. To precipitate proteins, 100 μL of aqueous trichloroacetic acid (15% V/V) was added. The samples were then briefly vortexed, placed in an ice bath for 5 minutes, then centrifuged for 10 min at 14,000•g and 4 ^o^C. The supernatant (100 μL) was transferred to a screw-cap 500 μL microcentrifuge tube and 50 μL of 2-thiobarbituric acid (0.375% w/v) as a solution in HCl (0.25 M) was added. The tubes were capped, vortexed, and placed in a dry bath incubator at 100 ^o^C for 60 min. They were then cooled on ice and centrifuged for 5 minutes at 14,000•g and 4°C, before the resulting derivatized solution (60 μl) was transferred to a 250 μL microinsert HPLC vial for analysis.

For calibration, a standard stock solution was prepared daily by diluting 1.00 mL of 1,1,3,3-tetraethoxypropane (TEP) (25 μL into 1.00 mL of anhydrous ethanol; 100 mM) by a factor of 1:100 with ultrapure water. Standards were prepared through serial dilution of the stock standard (1 mM) with ultrapure water to obtain concentrations of 20, 15, 10, 5, 2.5, 1.25, and 0 (blank) μM TEP. Quality control (QC) samples were prepared by pooling 5 μL of each derivatized sample in a single HPLC vial. The QC sample was injected every twenty sample injections, and again at the end of the series run. Standards were measured from triplicate injections. Samples were prepared in duplicate and measured from duplicate injections.

### FRAP and HASC analyses by spectrophotometry

#### System settings

All spectrophotometric assays for microplate analyses were conducted in standard flat bottomed 96 well plates (P/N 82.1581 Sarstedt AG & Co. KG, Nümbrecht, Germany) Analyses was carried out on an iMark Filter absorbance microplate reader with computer control and data analysis by Microplate Manager 6 Software (Bio-Rad Laboratories Canada Ltd., Mississauga, ON).

#### Uric acid independent FRAP analysis for 5 μL of plasma

The FRAP assay protocol was adapted from [39]. The amount of uricase added was reduced to 5 μL of 1U/mL after calculating the expected physiological range of uricase in plasma and the theoretical rate at which the uric acid could be removed by uricase. 1U/mL uricase was compared to 10U/mL of uricase as used by [39], with no observable difference in FRAP levels.

A 5 μL aliquot of plasma or standards (see below) was mixed with 5 μL ultrapure water-containing uricase (1.00 U/mL) in a well of a 96 well plate and incubated for 5 min at room temperature on a 2D shaker. Then, 200 μL of FRAP reagent was added to each well. After a 30 min incubation at room temperature with regular shaking in the spectrophotometer, absorbance at 593 nm was read *vs*. reagent blank. Calibration curves were generated from a daily prepared standard stock FeSO_4_ solution (30mM), serially diluted to 3.0 mM, 2.0 mM, 1.0 mM, 0.5 mM and 0 mM (blank) using ultrapure water. FRAP reagent was freshly prepared each day by mixing 20.0 mL acetate buffer (pH 3.6, 300 mmol/L), 2.00 mL 2,4,6-Tris(2-pyridyl)-s-triazine (TPTZ) (10 mmol/L) in HCl (40 mmol/L) and 2.00 mL FeCl3 (20 mmol/L). All analyses were conducted in duplicate.

#### HASC analysis for 1 μL of plasma

An aliquot of plasma was added to a well of a 96 well plate containing 100 μL of ultrapure water and the plate was briefly vortexed on a 2D shaker. Then, 100 μL of oxidant solution (for samples) or standard solutions (see below) were added, followed by a 10 min incubation at room temperature on a 2D shaker. After that, 20 μL of a stock N,N-dimethyl-p-phenylenediamine (NPD) solution (83 μL diluted to 50mL in anhydrous ethanol) was added to every well, followed by a 1 min incubation on the 2D shaker, prior to reading the absorbance of the wells at 515 nm.

NPD solution was freshly prepared each day and protected from daylight. The oxidant solution was prepared daily by diluting 10% sodium hypochlorite by a factor of 500 and adjusting the pH to 6.2 using 0.6 M sulfuric acid. The concentration of hypochlorous acid prepared was determined by measuring the absorbance at 292 nm in a 1 cm pathlength quartz cuvette and using the Beer-Lambert law with an absorptivity coefficient of 350 L mol^-1^ cm^-1^. Calibration curves were generated by serial dilutions of the oxidant solution to factors of 2X, 4X, 8X, 16X, 32X with ultrapure water. The reagent blank was prepared using ultrapure water. All sample measurements were run in duplicate.

### Statistical analyses

The three developed methods were assessed for intra and inter day variability using relative standard deviations (%RSD) and repeatability (*r*) calculation. We used the ‘rpt’ function in the R package rptR (version 3.4.3), which estimates repeatability of the samples’ readings using a linear mixed-effects model framework [47]. Number of bootstraps and permutations were set at 1000 each.

Very few studies assessed the stability of the MDA-TBA_2_ adduct once prepared and in queue waiting for HPLC analysis. We compared the prepared QC sample’s wait time to its relative MDA value using a linear regression. Also, a possible day-to-day drift in reagents efficiency was investigated by comparing the QC value to the mean of all the individuals assessed in each day, using a linear regression.

## Results

**Fig 1:**
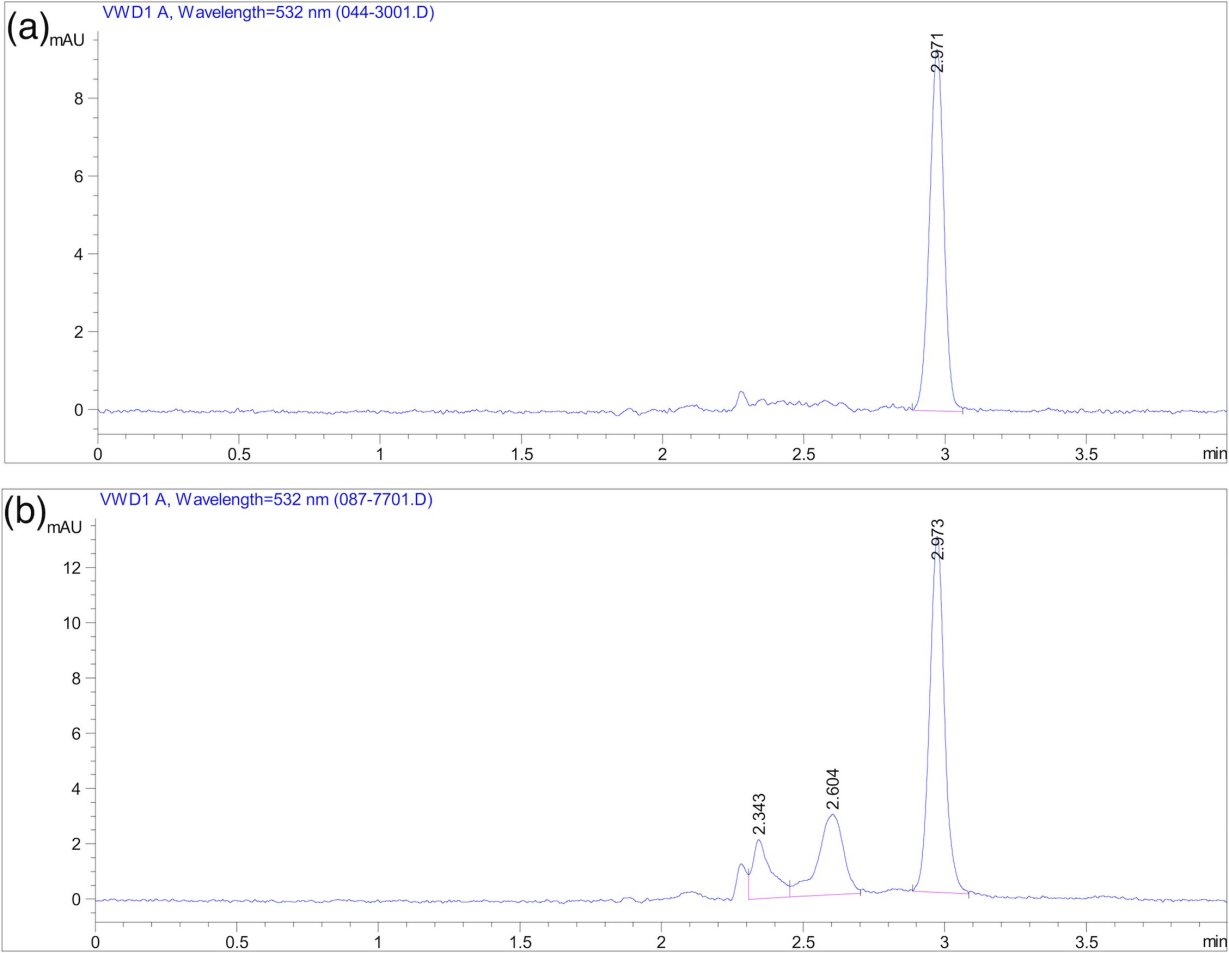
(a) HPLC chromatogram of 5 μM MDA standard, (b) HPLC chromatogram of plasma sample from an eastern chipmunk showing separation of interfering substances and MDA.

**Fig 2:**
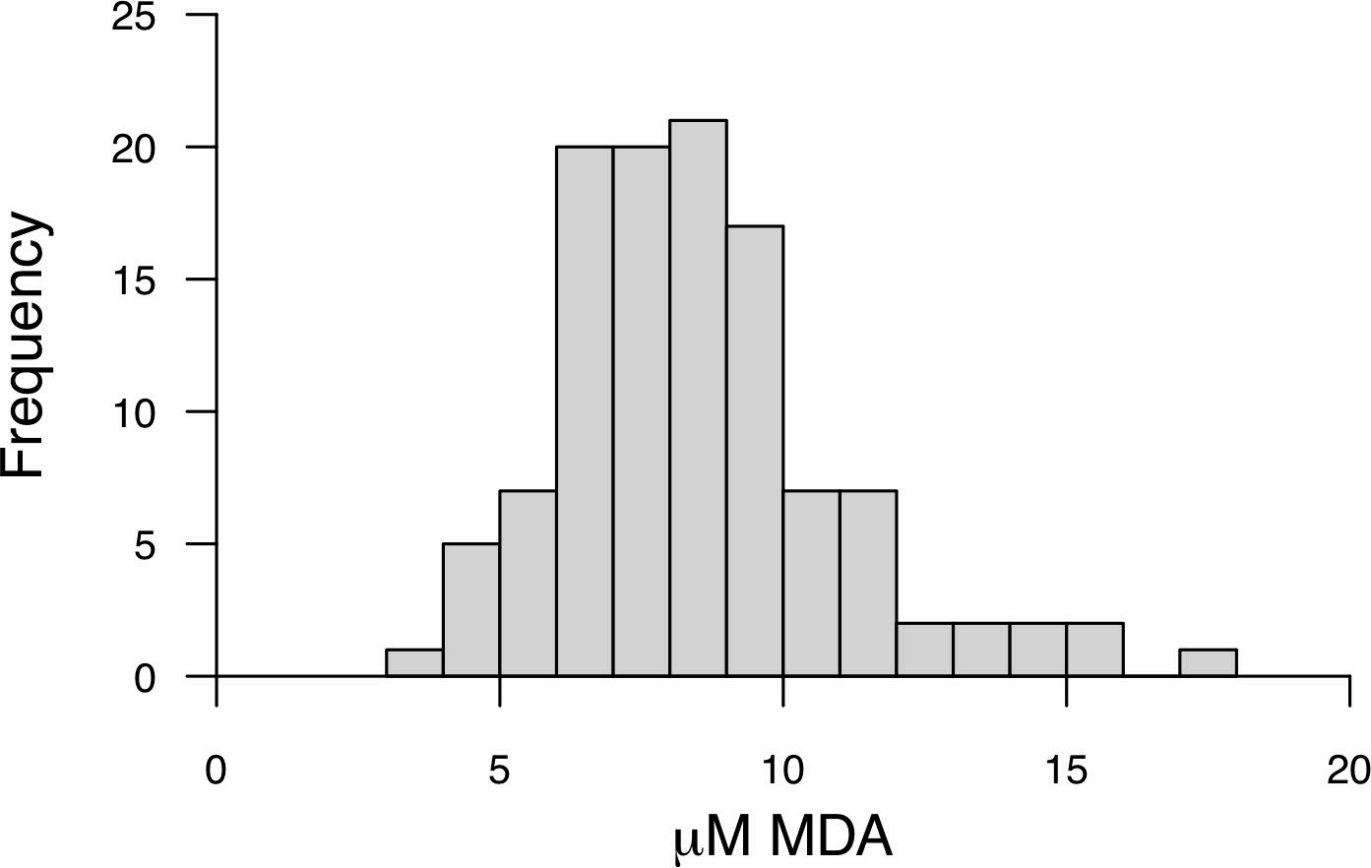
Distribution of MDA concentrations found in a population of wild eastern chipmunks (*Tamias striatus*).

Analysis of the MDA standard using the method developed yielded a single HPLC peak at 3.00 ± 0.05 min (Fig 1). The linearity of the calibration curve from 1.25–20 μM was high using this method (%RSD = 0.32, n = 7). The LOD and LOQ were 0.06 and 0.125 μM respectively. The inter-assay variation (%RSD = 9.31, n = 48) and repeatability (*r* = 0.82, n = 48, C.I. = 0.73–0.89) were representative of the laboratory measurement error, as well as differences in capillaries, as each technical replicate was prepared from a separate capillary from the blood sampling of an individual. We obtained no significant increase for QC samples over time (ß = 0.0195 μM per hour, r^2^ = 0.017, n = 8). The QC quantifications compared to the mean of all the individuals assessed in each day revealed that there is little effect on the results from different days of analysis (slope = 0.99, r^2^ = 0.98, n = 9). The MDA values obtained for individuals ranged from 4.4 to 15.6 μM (Fig 2).

**Fig 3:**
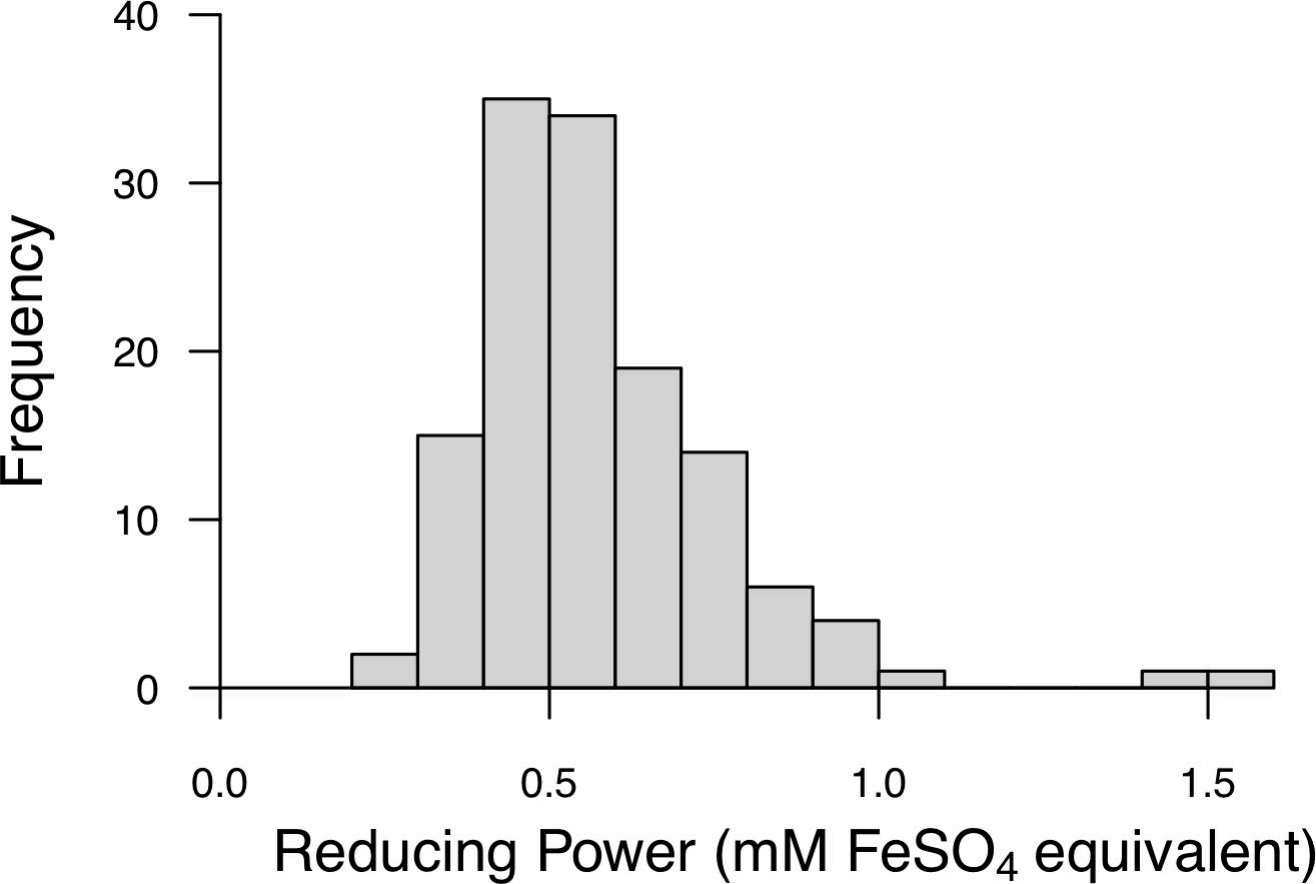
Distribution of FRAP assay values found in a population of wild eastern chipmunks (*Tamias striatus*).

For the developed uric acid independent FRAP assay, the linearity of the calibration curve, from 0.5–3 mM FeSO_4_, was high (%RSD = 2.50, n = 7). The inter-assay variation (%RSD) was 5.82 (n = 57) and repeatability was 0.95 (n = 57, CI: 0.92–0.97). The assay range for individuals was from 0.3 to 1.5 mM FeSO_4_ (Fig 3).

**Fig 4:**
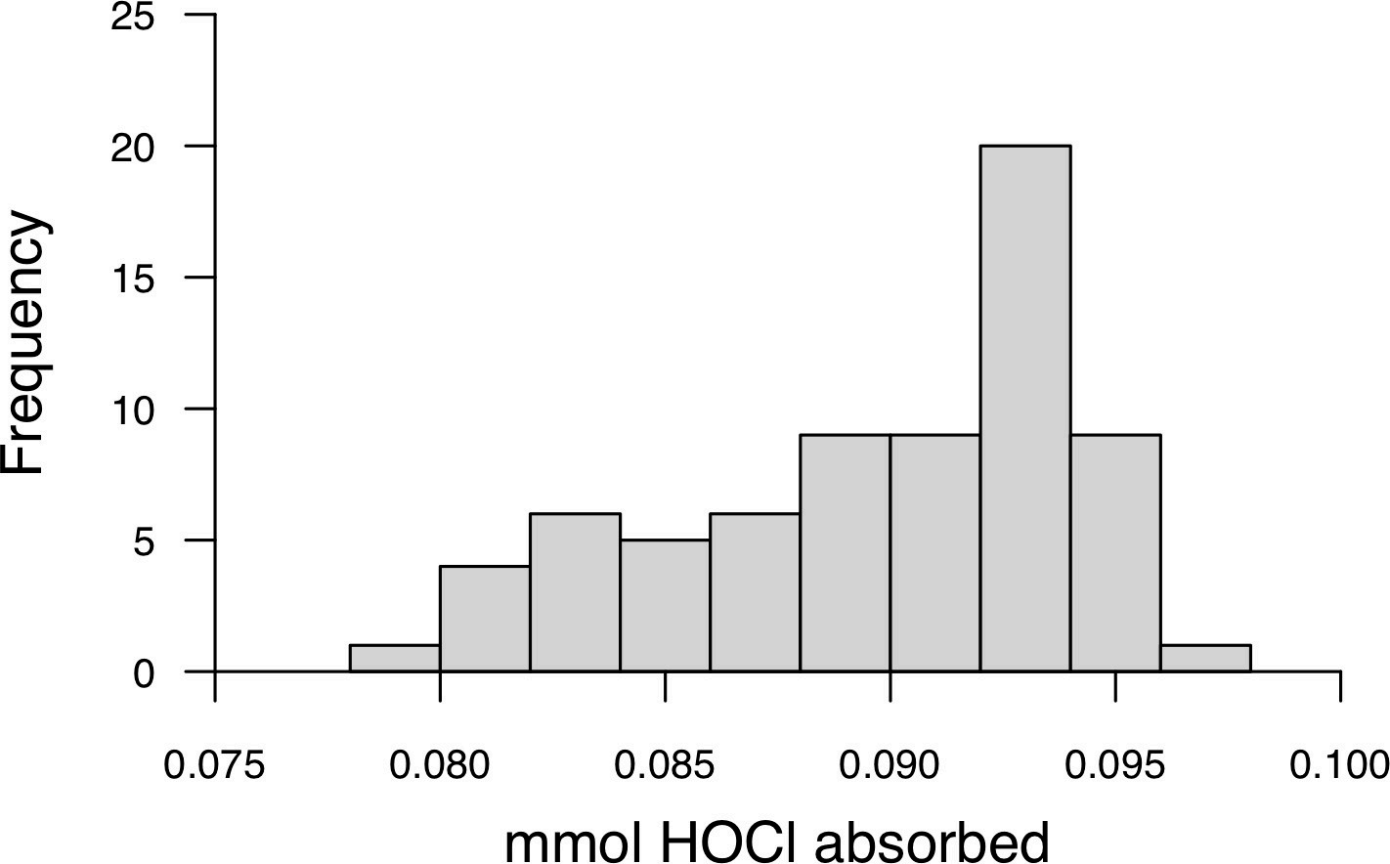
Distribution of hypochlorous acid shock capacities for 1 μL plasma found in a population of wild eastern chipmunks (*Tamias striatus*).

The linearity of the calibration curve for the adapted HASC assay, ranging from 0.03–0.5 mM HOCl, was high (%RSD = 3.64, n = 4). The inter-assay variation (%RSD) was 2.03 (n = 32) and repeatability was 0.79 (n = 32, CI: 0.64–0.89). The assay range for individuals was from 82 to 96 μmol HOCl consumed per microliter of plasma (Fig 4).

## Discussion

Several methods have been developed and modified for the measurement of oxidative stress in animal studies. However, these methodologies often require large blood volumes and are potentially biased by interfering substances. We report methodologies for three markers that can be used jointly to assess oxidative profile of small animals, requiring less than 55 μL of plasma to assay the three markers in duplicate. Despite reducing the required volumes, our methods show the average error level reported in other biological studies. We reported a repeatability of 0.82 for MDA, while previous ecological studies reported repeatabilities averaging 0.82 (ranging from 0.62 to 0.98; [15,38,48–50]. For FRAP and HASC, our reported inter-assay %RSD are 5.82 and 2.03, respectively, whereas other studies reported inter-assay coefficients of variation averaging 3.94% for FRAP (ranging from 3.00% to 5.50%; [8,11,39,45,51]) and 5.16% for the OXY adsorbent test^TM^ kit (ranging from 1.96% to 9.73% for the OXY adsorbent test^TM^ kit; [11,45,52–54].

For MDA analysis, we found that the most efficient separation and elution was with a 70:30 isocratic mixture of water with 0.1% TFA:acetonitrile. Using this method, we were able to reduce the elution time to three minutes and subsequently the runtime to four minutes with no need for equilibration between runs. In this manner, we improved the efficiency of the method in order to process more samples in a given period of time. We estimate that one could process 40 individuals and 4 calibration curves in a single day using the presented method.

Through our method development process, we noted that prepared samples were stable in the HPLC queue for up to 24 hours. Thus a larger number of samples could be prepared in one day and loaded into the HPLC system queue to run. We carried out all sample preparation in single 500 μL microcentrifuge tubes, however using microplate sample preparation technologies could be beneficial to analyse a larger number of samples. In this way, samples could be prepared and processed with less manipulation. Moreover, an autosampler equipped to sample directly from the wells of a microplate would further increase the streamlining of the method.

Some research has reported [39] a simple and rapid method for the determination of uric-acid independent FRAP levels. They used 200 μL of plasma and 10 U/mL uricase for the determination of uric acid independent FRAP values. We modified this approach to obtain a method that used 5 μL of plasma, which make the assay more appropriate for small animal studies. Also, reducing the amount of required uricase to 5 μL at 1U/mL substantially decreased the cost of the assay. In addition, we modified the method to be suitable for microplate measurement, therefore making the method amenable for large scale studies.

Our HASC assay method was developed from the OXY-Adsorbent kit^TM^ by Diacron International (Grosseto, Italy). The kit’s protocol requires 10 μL of plasma for each reading. We reduced the volume of plasma needed to 1 μL and kept the method adapted to a microplate assay. This allows a better control the incubation times, yielding more accurate reading, while minimizing laboratory error and maximizing throughput. Using our method, we estimate that one would be able to process 200 individuals in duplicate and 10 calibration curves in a single day. The simple and inexpensive NPD dye and commercially available bleach needed for this method have dramatically reduced the cost of the assay to a fraction of the kit’s cost.

Because we decreased the price and tissue volumes required for the three presented markers, more future studies should be able to use them jointly to thoroughly address an individual’s oxidative and antioxidant status. Although HASC and FRAP are two total antioxidant markers, they do not focus on the same antioxidant group in the cellular machinery. FRAP measures the effect of molecules with an active reducing power, whereas HASC also measure the contribution of passive sacrificial molecules such as proteins like albumin, amino acids, nucletotides, etc. [55–57]. These sacrificial elements can contribute to absorb oxidative damage and indirectly prevent or delay the degradation of other cellular structures that are more important to maintain cellular integrity. Although they are not part of the oxidative stress regulation, it is argued that these sacrificial elements could play a major role in antioxidant protection because of their quick turn over [5,14,58]. Thus, these two general antioxidant assays could depict two complementary components of an individual’s antioxidant status [11,45].

Overall, we have presented three improved methodologies for the measurement of oxidative stress in small animal plasma samples. We have improved the methods by lowering plasma volumes required, increasing analysis efficiency and throughput, and removing prominent interfering signals in the analyses. All three tests that we have developed have shown a high repeatability. With these improved techniques, we hope to provide ecologists tools to better understand the factors which influence individuals’ and populations’ oxidative stress trends.
